# Microglia P2Y6 receptor is related to Parkinson’s disease through neuroinflammatory process

**DOI:** 10.1186/s12974-017-0795-8

**Published:** 2017-02-20

**Authors:** Xiaodong Yang, Yue Lou, Guidong Liu, Xueping Wang, Yiwei Qian, Jianqing Ding, Shengdi Chen, Qin Xiao

**Affiliations:** Department of Neurology and Institute of Neurology, Ruijin Hospital, Shanghai Jiao Tong University School of Medicine, 197 Ruijin Er Road, Shanghai, 200025 China

**Keywords:** Parkinson’s disease, P2Y6 receptor, Microglia, Neuroinflammation

## Abstract

**Background:**

Microglia in the central nervous system (CNS) were reported to play crucial role in neurodegeneration. Previous studies showed that P2Y6 receptor (P2Y6R) mainly contributed to microglia activation and phagocytosis in CNS. However, the level of P2Y6R in Parkinson’s disease (PD) patients is unclear. Therefore, we measured the level of P2Y6R in PD patients and speculated whether it could be a potential biomarker for PD. Given on the basis that P2Y6R was higher in PD patients, we further explored the mechanisms underlying P2Y6R in the pathogenesis of PD.

**Methods:**

We tested the expression level of P2Y6R in the peripheral blood mononuclear cells (PBMCs) among 145 PD patients, 170 healthy controls, and 30 multiple system atrophy (MSA) patients. We also used a lipopolysaccharide (LPS)-stimulated microglial cell culture model to investigate (i) the effects of LPS on P2Y6R expression with western blot and RT-PCR, (ii) the effects of LPS on UDP expression using HPLC, (iii) the effects of UDP/P2Y6R signaling on cytokine expression using western blot, RT-PCR, and ELISA, and (iv) the signaling pathways activated by the P2Y6R involved in the neuroinflammation.

**Results:**

Expression levels of P2Y6R in PD patients were higher than healthy controls and MSA patients. P2Y6R could be a good biomarker of PD. P2Y6R was also upregulated in LPS-treated BV-2 cells and involved in proinflammatory cytokine release through an autocrine loop based on LPS-triggered UDP secretion and accelerated neuroinflammatory responses through the ERK1/2 pathway. Importantly, blocking UDP/P2Y6R signaling could reverse these pathological processes.

**Conclusions:**

P2Y6R may be a potential clinical biomarker of PD. Blocking P2Y6R may be a potential therapeutic approach to the treatment of PD patients through inhibition of microglia-activated neuroinflammation.

## Background

Microglia are the major cells involved in immune function within the CNS, and accumulating evidence suggests that microglia act as key players in the initiation of various CNS diseases, including Parkinson’s disease (PD) [1–3]. Postmortem analyses of PD patients and experimental animal studies have indicated that the activation of glial cells and increased proinflammatory factor levels are common features in PD brains [4, 5]. A better understanding of the mechanism of the regulation of microglial function is crucial for developing effective therapeutics for PD. However, the trigger for inducing microglial release of proinflammatory mediators in PD remains to be elucidated.

Nucleotides have been explored as inducers of brain inflammation [6]. In the context of chronic brain inflammation, neurons can release both adenine (such as ATP) and uracil nucleotides (such as UTP and UDP) into the extracellular space upon physiological activity and in response to pathological stimuli and cellular damage, which affects the motility of adjacent cells, including microglia [7, 8]. The roles of adenine nucleotides, such as ATP, in inflammatory processes have been intensively studied [9]. Nucleotide receptors are normally divided into two subfamilies: the ionotropic receptor (P2X) family and the metabotropic receptor (P2Y) family. The activation of P2Y receptors plays important roles in the nervous system. The activation of P2Y1 receptor can modulate neuronal activity and neuronal fiber outgrowth [10, 11], and P2Y12 receptor is involved in microglial migration driven by ATP [12]. There is evidence of a prominent role of P2Y2 receptor in Alzheimer’s disease pathology [13, 14].

As a member of the P2Y receptor family, the purinoceptor P2Y6 receptor (P2Y6R) is widely distributed in various tissues, including the placenta, spleen, thymus, small intestine, blood, heart, blood vessels, and brain [15]. P2Y6R plays an important role in immunoinflammation in the peripheral system. UDP, known to be a specific agonist of P2Y6R, has been demonstrated to mediate the secretion of C-X-C motif chemokine ligand 8 (CXCL8) and macrophage inflammatory protein-3 (MIP-3a) in monocytes and in intestinal and lung epithelial cells [16–18]. P2Y6R is highly expressed on T cells that infiltrate inflamed colonic tissues but is absent in T cells of the unaffected bowel [19]. P2Y6R also participates in toll-like receptor (TLR) 1/2-induced neutrophil migration by regulating IL-8 secretion [20]. Moreover, P2Y6R activation potentiates proinflammatory responses in macrophages [21]. In the CNS, a number of studies have demonstrated that damaged neurons can release UDP, which contributes to the microglial phagocytosis of dying neurons [22, 23]. In addition, UDP induced the expression of chemokines via a P2Y6R-dependent mechanism in brain microglia and astrocytes [24]. However, the role of P2Y6R in the CNS, especially in microglia-associated processes, requires further investigation, and whether its level is abnormal in sporadic PD patients remains to be identified.

Here, we found that P2Y6R levels were elevated in the peripheral blood mononuclear cells (PBMCs) of PD patients and, therefore, P2Y6R could be a potential clinical biomarker of PD. UDP/P2Y6R signaling was involved in the neuroinflammation mediated by microglia, and blocking P2Y6R could be a therapeutic approach for treating PD patients via the inhibition of microglia-activated neuroinflammation.

## Methods

### Patients and samples

In our study, 145 PD patients, 170 control subjects, and 30 possible multiple system atrophy (MSA) patients were recruited from the Department of Neurology, Ruijin Hospital affiliated to Shanghai Jiao Tong University School of Medicine. The clinical diagnosis of PD was established by two independent senior movement disorder specialists using the United Kingdom Parkinson’s Disease Society Brain Bank clinical diagnostic criteria [25]. The diagnosis of possible MSA was made according to the second consensus statement on the diagnosis of MSA [26]. The clinical features of the PD patients were collected, and PD symptoms were evaluated using the Unified Parkinson’s Disease Rating Scale (UPDRS) and the Hoehn and Yahr (H-Y) staging modified version. The study was approved by the Ethics Committee of Ruijin Hospital affiliated to Shanghai Jiao Tong University School of Medicine, and written informed consent was obtained from all participants. Venous whole blood samples (4–5 ml) were collected from the individuals of the three groups on an empty stomach in the morning and mixed thoroughly with EDTA to prevent coagulation. Blood samples were collected in tubes containing EDTA. Then, the peripheral blood mononuclear cells (PBMCs) were lysed with 1 ml TRIzol reagent, and the total RNA was harvested as described by the manufacturer’s protocol. Quantitative real-time PCR (qRT-PCR) was performed using 4 μg of total RNA as a template for the reverse transcription reaction using a TakaRa 1st-strand kit. Reactions were performed according to the manufacturer’s protocol. Complementary DNA (cDNA) synthesized from each sample was subjected to real-time PCR assays with specific primers and a SYBR@Premix Ex TaqTM qPCR SuperMix (Takara). The sequences of primers were as follows: human-P2Y6R: forward 5-GCT AAC TCT TGG CTC CCG AAC A-3, reverse 5-GCG GGA CGT GCA GAT CTG GGT A-3; GAPDH: forward 5-CGG AGT CAA CGG ATT TGG TCG TAT-3, reverse 5-AGC CTT CTC CAT GGT GGT GAA GAC-3. The two-step amplification protocol consisted of denaturation for 30 s at 95 °C and then followed by 40 cycles of 95 °C for 5 s and 60 °C for 60 s. This process was performed using a ABI7500 real-time PCR machine. The RNA quantities of target genes were calculated using the Ct method. The Ct values of P2Y6R amplification were normalized to those of GAPDH amplification.

### Materials

Uridine-5’-diphosphate sodium (UDP), MRS2578, lipopolysaccharide (LPS) (*Escherichia coli* 011:B4), apyrase, dimethyl sulfoxide (DMSO), and MTT were purchased from Sigma Chemical Co. (St. Louis, MO). U0126, SB203580, and SP600125 were obtained from Cell Signaling Technology (Danvers, MA). Optim-MEM and Lipofactamine 2000 were obtained from Invitrogen (Carlsbad, CA). The small-interfering RNA (siRNA) of P2Y6R was provided by GenePharma (Shanghai, China).

### Cell culture and transfection

Primary microglia were derived from male neonatal C57BL/6 mice (1-day old), using the “shaking off” method. Briefly, cerebral cortices were devoid of meninges and blood vessels, dissected in Hank’s salt (HBSS) and trypsinized with 0.25% trypsin-EDTA for 30 min at 37 °C. The mixed glial culture was incubated in DMEM/F-12 containing 10% fetal bovine serum (FBS) and 50 U/ml penicillin/streptomycin (PS) and seeded at 20 × 10^6^–25 × 10^6^ cells per 175 cm^2^ flask coated with poly-l-ornithine (Gibco). After 2 weeks of culture, flasks were shaken and the media were collected and centrifuged at 1000 rpm for 5 min to obtain a pellet of microglia. Isolated microglia were allowed to attach to plates for 3 to 4 h, and unattached cells were removed by changing the medium. Microglial cultures were determined to be 95 to 98% pure, as assessed by staining for Iba-1.

BV-2 cells, SH-SY5Y cells, and N2a cells were routinely cultured in Dulbecco’s modified Eagle’s medium (DMEM) supplemented with 10% FBS and 100 U/ml PS. PC 12 cells were cultured in DMEM medium supplemented with 5% horse serum and 5% FBS. The cells were plated 24 h prior to transfection at a confluency of 70 to 80%. The cells were transfected with P2Y6R or control siRNA (scramble) by using Lipofectamine 2000 according to the manufacturer’s instructions. The P2Y6R siRNA sequences were as follows:

- First: 5-CCCUACUUAUCUAUAACUATT-3, 5-UAGUUAUAGAUAAGUAGGGTT-3;
- Second: 5-CUGGCUGCAAUGUGUUUGUTT-3, 5-ACAAACACAUUGCAGCCAGTT-3,
- Third: 5-GGUCAAUUCAUGCUUGUUATT-3, 5-UAACAAGCAUGAAUUGACCTT-3
- Scrambled siRNA: 5-UUCUCCGAACGUGUCACGUTT-3
- 5-ACGUGACACGUUCGGAGAATT-3

### Reverse transcription polymerase chain reaction

Cells were plated at a density of 3 × 10^5^ cells/well in six-well plates, as detailed above. Then, the cells were lysed with 1 ml TRIzol, and the total RNA was harvested as described by the manufacturer’s protocol. Reverse transcription polymerase chain reaction (RT-PCR) was performed using 4 μg of total RNA as a template for the reverse transcription reaction using a TakaRa 1st-strand kit. The cDNA was then used for PCR of the murine purinergic P2Y6R and cytokine genes using the following primers:

- P2Y6R:
- forward 5-GCA TGAGACAGACTCTCCGAGCA-3,
- reverse 5-ACAAAGCGGCAG GCGAGGTC-3;
- Inducible form of nitric oxide synthase (iNOS):
- forward 5-CTTCACCGGGTCAGAGCCACA GTCC-3,
- reverse 5-GAGCCAAGGCCAAACACAGCATAC-3; cyclooxygenase-2 (COX-2):
- forward 5-CAGCAAATCCTTGCTGTTCC-3,
- reverse 5-TGG GCAAAGAATGCAAACATC;
- Tumor necrosis factor-α (TNF-α):
- forward 5-ATGAGCACAGAA AGCATGATC-3,
- reverse 5-TACAGGCTTGTCACTCGAATT-3;
- Interleukin-6 (IL-6):
- forward 5-CCACTTCACAAGTCGGAGGCTT-3,
- reverse 5-CCA GCTTATCTGTTAGGAGA-3;
- Macrophage inhibitory protein-2 (MIP-2):
- forward 5-GCTCCTCAGTGC ACTGGTC-3,
- reverse 5-GTTAGCCTTGCCTTTGTTCAGTATC-3;
- β-actin:
- forward 5-AATCCTGGCATCCATGAAACTAC-3,
- reverse 5-TCTGCTGGAAGGTGGACAGTGAG-3.

The PCR reaction protocol was as follows: denaturation at 95 °C for 5 min; followed by 25 cycles at 95 °C for 30 s; P2Y6R, iNOS, COX-2, IL-6, and TNF-α at 54 °C and MIP-2 and β-actin at 58 °C for 30 s, 72 °C for 30 s, and finally, extension at 72 °C for 5 min. The PCR products were separated by 2% agarose gel electrophoresis and visualized via ethidium bromide staining.

### Immunofluorescence analysis

To examine the morphology change of primary microglia cells, we pretreated primary microglia cells with apyrase (1 U/ml) and MRS2578 (5 μM) for 30 min and then treated with LPS (100 ng/ul) for 12 h. The cells were fixed with a fresh formaldehyde solution for 20 min followed by permeabilization with 0.15% Triton X-100 in PBS. Cells were then incubated overnight at 4 °C with rabbit anti Iba1 antibody (019–19741, 1:200; Wako), followed by washing in PBS 3 × 10 min. Then, the slices were incubated with the Alexa Flour 594 goat anti-rabbit IgG secondary antibody (ab150080, 1:500; Sigma) for 1 h. Finally, cells were incubated with DAPI (10236276001, Roche; 1:20000) for 15 min. Images were analyzed with a phase contrast fluorescence microscope (Nickon Eclipse E400). Experiment was performed in triplicate.

### Western blotting

Cells were washed in ice-cold phosphate-buffered saline (PBS) three times and lysed in RIPA lysis buffer (50 mM Tris–HCl, pH 8.0; 1% NP-40; 0.5% sodium deoxycholate; 150 mM NaCl; 0.1% SDS) containing protease and phosphatase inhibitor cocktails (Roche) and 1 mM phenyl-methylsulphonyl fluoride (PMSF). Protein content was determined using a Micro-BCA Protein Assay (Thermo Scientific). Equal amounts of protein (30 μg) were loaded per lane and separated by 10% SDS-PAGE. The proteins in the gels were transferred to Immobilon polyvinylidene difluoride (PVDF) membranes (Santa Cruz, CA, USA), and the membranes were subsequently blocked in TBST (10 mM Tris–HCl, pH = 8.0; 150 mM NaCl; 0.05% Tween-20) containing 5% nonfat milk. Anti-P2Y6 receptor polyclonal goat antibody (sc-15217, 1:1000, Santa Cruz), anti-phospho-p38 (#4511); anti-phospho-ERK1/2 (#4370), and anti-phospho-JNK (#4668) antibodies; the corresponding total-p38 (#9219), total-ERK1/2 (#9102), and total-JNK (#9528) antibodies; and anti-cleaved caspase-3 (#9661) antibody (Cell Signaling Technology) were used at a dilution of 1:1000 in 3% milk/TBST. The levels of iNOS and COX-2 were determined using anti-iNOS antibody (sc-69190, 1:500; Santa Cruz) and anti-COX-2 antibodies (160106, 1:800; Caymen Chemical). All the primary antibodies were incubated overnight at 4 °C. The immunoreactive bands were visualized using secondary antibodies conjugated to horseradish peroxidase (Santa Cruz, CA, USA) and chemiluminescent detection methods (Amersham Company). To confirm equal protein loading, the membranes were probed with antibodies recognizing the cytosolic protein, β-actin (A1978, 1:2500; Sigma-Aldrich). The data shown are representative of at least three separate experiments.

### High-performance liquid chromatography

Cultured BV-2 cells were stimulated with and without 200 ng/ml LPS for 12 h, and the supernatant was then collected and cleared of cellular debris. Detection of UDP in the cell culture medium was determined by reverse-phase high-performance liquid chromatography (HPLC) (Varian Vista series) using an Agilent Zorbax 300SB-C18 column (300 A), as described previously [27]. Cell medium samples (200 μl) or standard samples were injected. The retention time of UDP released by the BV-2 cells was compared with standard samples, and the peaks were identified by comparing their retention times with those of co-eluted standards. Quantification was performed by integration of the peak areas and compared with those produced by standards of known concentrations prepared in the same solvent.

### ELISA

The conditioned medium collected from the BV-2 cells treated with LPS was evaluated for the production of MIP-2 cytokines using ELISA (BD Biosciences, San Jose, Calif.). ELISA was performed as recommended by the manufacturer. The optical density of each well was determined using a microplate reader at 405 nm, and the amounts of cytokines were calculated from the standard curve.

### MTT assay

Cell viability was measured by an MTT assay. The neuronal cells (SH-SY5Y, N2a, and PC12 cells) were plated in 96-well plates. After treating with the conditioned medium separated from the BV-2 cells for 24 h, MTT solution at a final concentration of 5 mg/ml was added to each well, and the plates were incubated for another 4 h. One hundred fifty microliters of DMSO was then added to solubilize the MTT tetrazolium crystals. Finally, the optical density was determined at 570 nm.

### Statistical analysis

Analyses were conducted with t GraphPad Prism 6.0 Software. Data were expressed as the mean ± SD and were compared by using Student’s *t* test between two groups and one-way or two-way ANOVA with Bonferroni’s correction for multiple group comparison. Pearson’s correlation coefficient was chosen for correlation analyses.

## Results

### P2Y6R mRNA in PBMCs from PD patients is increased compared with healthy controls and MSA patients

**Fig. 1:**
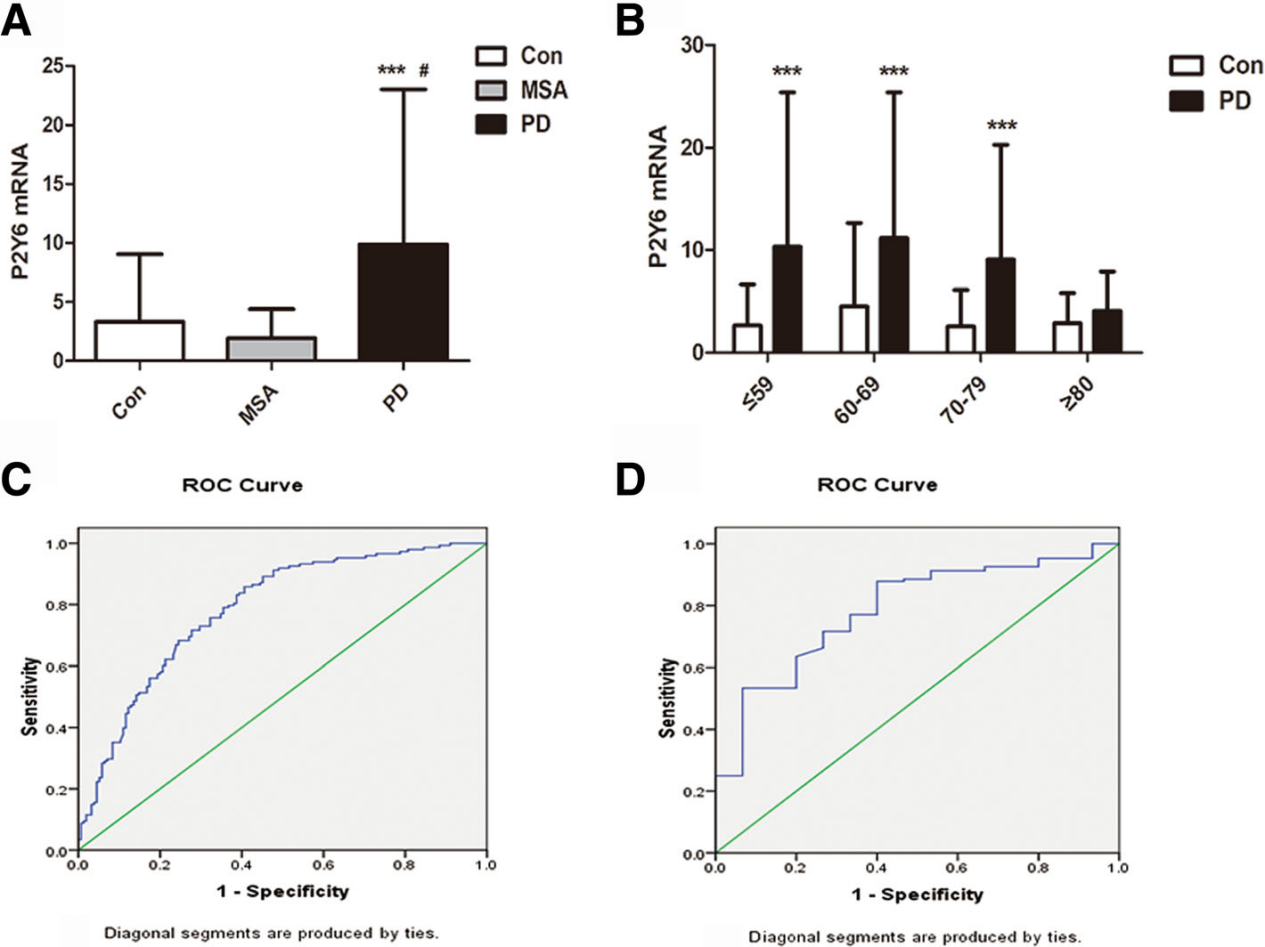
Increased P2Y6R mRNA levels in PBMCs from PD patients. **a** mRNA levels of P2Y6R in PBMCs of healthy controls, MSA, and PD patients. The gene expression of P2Y6R was normalized to those of GAPDH expression. **b** Expression of P2Y6R mRNA among different age groups of PD patients and control subjects. **c** The ROC curve of PD patients and healthy controls. **d** The ROC curve of PD patients and MSA patients. Data are shown as the mean ± SD, *n* = 170 for healthy controls, *n* = 145 for PD patients, and *n* = 30 for MSA patiens. ***p < 0.001 vs Con, ^#^ *p* < 0.001 vs multiple system atrophy (MSA)

We measured the levels of P2Y6R messenger RNA (mRNA) via qRT-PCR in 145 PD patients, 170 normal controls, and 30 possible MSA patients. The subjects’ demographics are summarized in Table 1. The results showed that the relative amount of P2Y6R mRNA in the PD patients was significantly higher than that in the healthy controls and MSA patients, while there was no difference between healthy controls and MSA patients (Fig. 1a). Furthermore, we compared the P2Y6R mRNA levels among different age groups. P2Y6R mRNA expression levels in PD patients in the <60, 60–69, and 70–79 age groups were approximately 3.9, 2.5, and 3.5 times greater compared to the respective age groups of the healthy controls (Fig. 1b). However, P2Y6R mRNA expression was not significantly different between PD patients and healthy controls older than 80. In addition, P2Y6R mRNA expression was uncorrelated with gender, age of disease onset, disease duration, H&Y stage, UPDRS score, and drug administration (data not shown). We also plotted the receiver operating characteristic curve (ROC curve) according to the expression levels of P2Y6R mRNA in both PD patients and healthy controls, with the cutoff point being the presence of disease or not. The area under the curve was 0.785 (95% CI = 0.734–0.838), the diagnostic cutoff point was 2.15, and the diagnostic sensitivity and specificity were 85.8 and 59.4% (Fig. 1c). Regarding PD patients and MSA patients, the area under the ROC curve was 0.792 (95% CI = 0.778–0.959), the diagnostic cutoff point was 2.77, and the diagnostic sensitivity and specificity were 76.4 and 93.7% (Fig. 1d).

**Table 1:** The demographic characteristics of healthy controls, PD patients, and MSA patients

	Healthy controls *n* = 170	PD group *n* = 145	MSA group *n* = 30
Male, *n* (%)	95 (54.84%)	85 (60.81%)	16 (53.33%)
Age (year)	66.05 ± 9.01	66.05 ± 10.07	61.35 ± 5.91
P2Y6R 2^−ΔΔCt^	3.33 ± 5.71	9.88 ± 13.13	1.94 ± 2.46
Age (year)			
<60	2.67 ± 3.96 (*n* = 48)	10.32 ± 15.04 (*n* = 34)	
60–69	4.51 ± 8.17 (*n* = 62)	11.47 ± 14.19 (*n* = 60)	
70–79	2.56 ± 3.56 (*n* = 42)	9.07 ± 11.22 (*n* = 41)	
≥80	2.89 ± 2.92 (*n* = 18)	4.09 ± 3.78 (*n* = 10)

We concluded that P2Y6R expression levels are elevated in PD patients and have no relationship with the severity of disease and that P2Y6R expression might be a stable diagnostic biomarker for PD.

### LPS-activated microglia and upregulated P2Y6R expression

**Fig. 2:**
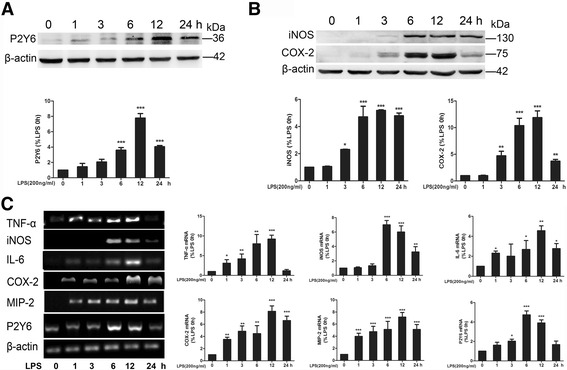
LPS-activated microglia and upregulated P2Y6R expression. **a** P2Y6R expression in BV-2 cells after LPS stimulation according to western blot. **b** iNOS and COX-2 levels after LPS stimulation according to western blot. **c** Inflammatory cytokine (TNF-α, iNOS, IL-6, COX-2, and MIP-2) and P2Y6R expression levels after LPS stimulation according to RT-PCR. The band intensity was quantified using studio lite imager and is presented relative to the level of β-actin. Data are shown as the mean ± SD of three independent experiments. *p < 0.05, **p < 0.01, ***p < 0.001 vs LPS 0 h

We investigated the expression of P2Y6R in an activated microglia cell model. We found that incubation of BV-2 cells with LPS increased both mRNA and protein levels of P2Y6R in a time-dependent manner, and the highest levels of mRNA were reached at 6 h and of protein at 12 h (Fig. 2a, c). The trends in the expression of various inflammatory cytokines (TNF-α, iNOS, IL-6, COX-2, and MIP-2 mRNA) were accord with the level of P2Y6R in LPS stimulated BV-2 cells. The relative amounts of TNF-α, iNOS, IL-6, COX-2, and MIP-2 mRNA were increased (Fig. 2c). LPS stimulation for 6 and 12 h also significantly upregulated the protein expression levels of COX-2 and iNOS (Fig. 2b). These findings suggest that P2Y6R expression was upregulated after microglia activation.

### UDP/P2Y6R signaling involved in the activation of microglia and the production of inflammatory cytokines

**Fig. 3:**
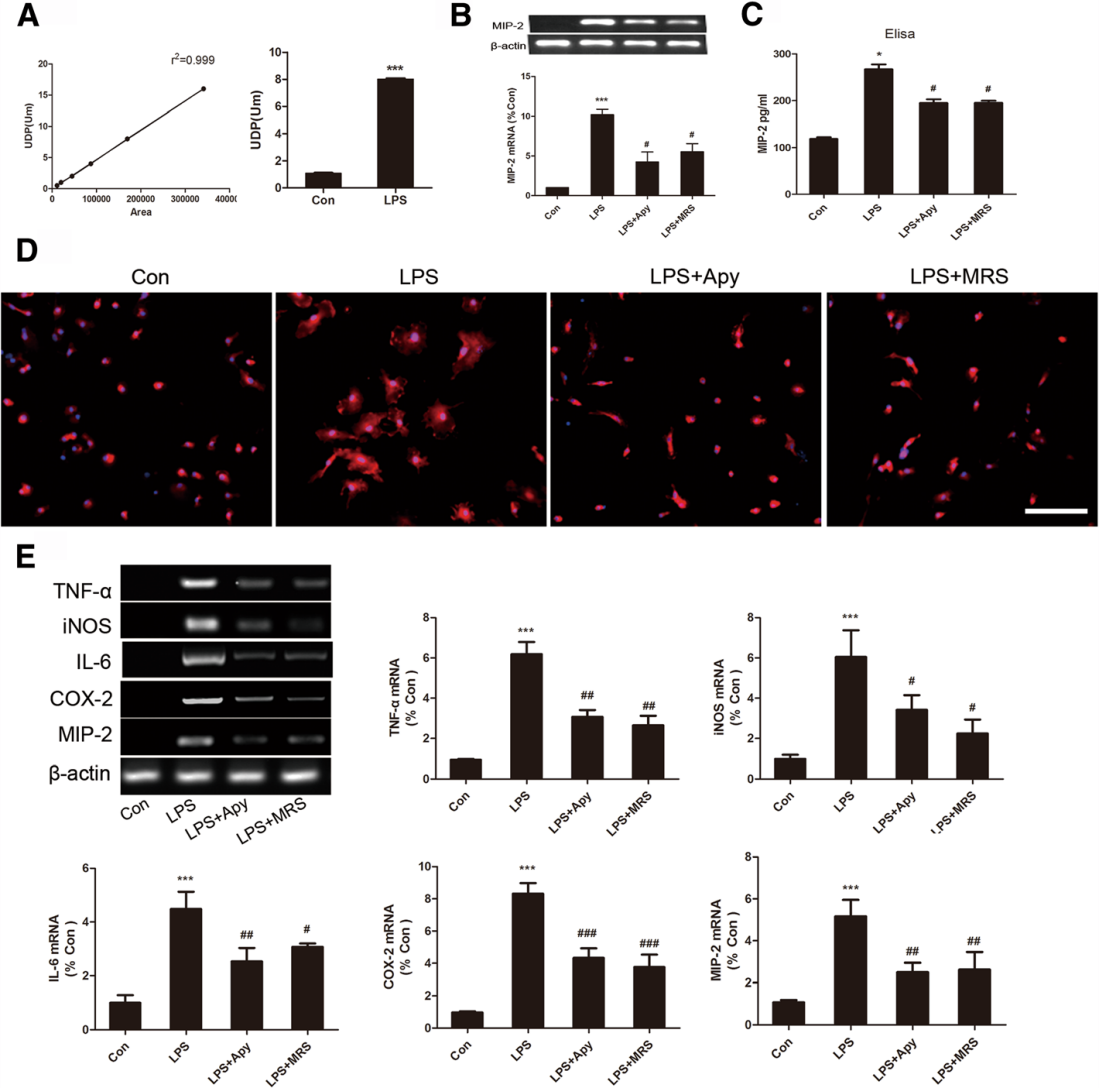
UDP/P2Y6R signaling involved in the activation of microglia cells and the production of inflammatory cytokines. **a** The standard curve and the UDP concentration in the LPS-treated and control supernatants of BV-2 cells based on HPLC. **b** mRNA levels of MIP-2 in LPS, LPS+Apy- and LPS+MRS-treated BV-2 cells. **c** ELISA of MIP-2 secretion. **d** Morphological changes in LPS-stimulated and Apy/MRS pretreated primary microglia cells. Representative immunostained images for primary microglia cells (Iba-1, *red*; DAPI, *blue*). *Scale bar* = 100 μm. **e** Representative RT-PCR showing the expression levels of inflammatory cytokines (TNF-α, iNOS, IL-6, COX-2, and MIP-2) from LPS-stimulated primary microglia cells pretreated with Apy/MRS. The band intensity was quantified using studio lite imager and is presented relative to the level of β-actin. Data are shown as the mean ± SD of three independent experiments. *p < 0.05, ***p < 0.001 vs Con, ^#^ *p* < 0.05, ^##^ *p* < 0.01, ^###^ *p* < 0.001 vs LPS. Apy (Apyrase, nucleotide hydrolase), MRS (MRS2578, P2Y6 inhibitor)

Because we have demonstrated that the change in P2Y6R expression was coordinated with inflammatory responses, we supposed that P2Y6R might play an important role in the LPS-induced production of inflammatory cytokines. UDP is known to be a specific agonist of the P2Y6 purinergic receptor, while LPS is not the direct agonist of P2Y6R. An obvious sine qua non of this hypothesis is that microglia can release UDP in response to LPS stimulation. We therefore investigated whether LPS increased the release of extracellular UDP from microglia. Cultured BV-2 cells were stimulated with and without LPS, and the supernatant was then collected for nucleotide assay based on HPLC. The amount of UDP in the LPS-treated supernatant was significantly higher than that in the untreated supernatant (Fig. 3a).

To examine the role of UDP/P2Y6R signaling in activated microglia-induced cytokine production, we pretreated the BV-2 cells with apyrase (1 U/ml) and MRS2578 (5 μM) for 30 min. Apyrase can hydrolyze tri- and di-phosphate nucleotides and therefore abrogate both ATP and UDP signaling [28, 29]. MRS2578 is the specific irreversible P2Y6R antagonist which could completely blocked P2Y6R [30, 31]. We found both apyrase and MRS2578 reduced MIP-2 mRNA expression levels induced by LPS stimulation (Fig. 3b). ELISA was also performed to quantify the impact of apyrase and MRS2578 on MIP-2 secretion. Both apyrase and MRS2578 significantly decreased the amount of MIP-2 released into the medium (Fig. 3c). To further validate the role of UDP/P2Y6R signaling in activated microglia-induced cytokine production, we turned to primary microglia cells. The morphology of microglia cells in the control group most frequently showed an ovoid shape. Primary microglia cells showed significant change in morphology after LPS treatment. Most of the LPS-stimulated microglia cells had a large and flat shape with other cells presenting a ramified morphology, while pretreated primary microglia cells with apyrase and MRS2578 can inhibit activation of the microglia as was seen by typically resembling morphology that seen in the control group (Fig. 3d). Confirming these results, we found that blocking UDP/P2Y6R signaling by apyrase and MRS2578 can reduce the inflammatory cytokines (TNF-α, iNOS, IL-6, COX-2, and MIP-2 mRNA) production in primary microglia cells induced by LPS (Fig. 3e).

**Fig. 4:**
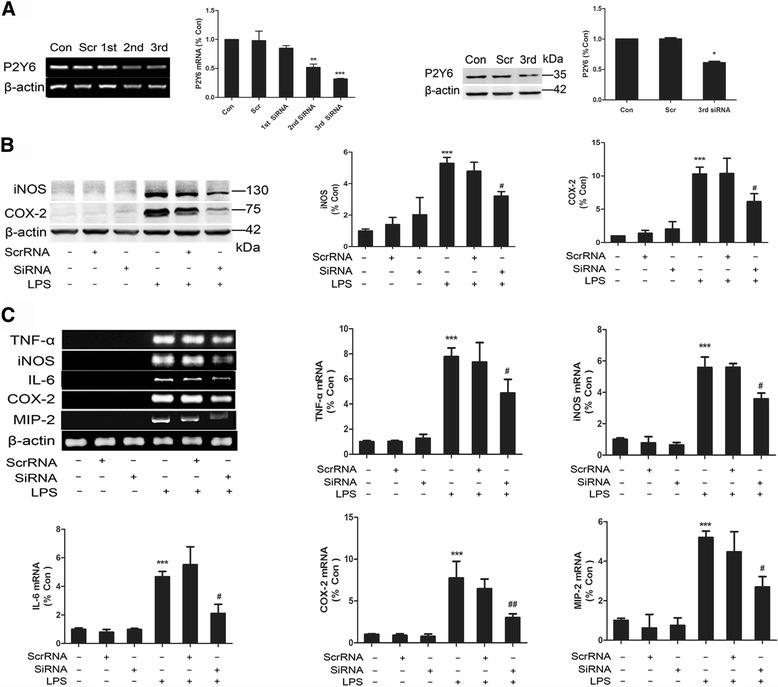
Knockdown of P2Y6R reduced the production of inflammatory cytokines. **a** RT-PCR and western blot analyses of P2Y6R from BV-2 cells transfected with P2Y6 siRNA. **b** Representative western blots showing iNOS and COX-2 levels from LPS-stimulated BV-2 cells transfected with P2Y6 siRNA. **c** Representative RT-PCR showing the expression levels of inflammatory cytokines (TNF-α, iNOS, IL-6, COX-2, and MIP-2) from LPS-stimulated BV-2 cells transfected with P2Y6R siRNA. The band intensity was quantified using studio lite imager and is presented relative to the level of β-actin. Data are shown as the mean ± SD of three independent experiments. *p < 0.05, **p < 0.01, ***p < 0.001 vs Con, ^#^ *p* < 0.05, ^##^ *p* < 0.01 vs LPS

To complement the experiments with antisense oligonucleotides, we also transfected BV-2 cells with P2Y6R siRNA to knock down P2Y6R expression. The interfering efficacy of P2Y6R mRNA and protein expression was confirmed by RT-PCR and western blot, respectively. We chose the siRNA with the best interference efficiency for further studies (Fig. 4a). Interestingly, we found that knocking down P2Y6R reduced the protein expression levels of iNOS and COX-2 in BV-2 cells induced by LPS (Fig. 4b) and the inflammatory cytokine production was also downregulated (Fig. 4c). These findings suggested that inhibition of UDP/P2Y6R signaling could partly prevent LPS-induced neuroinflammation.

### Knockdown of P2Y6R can increase neuronal cell viability and attenuate apoptosis upon exposure to conditioned medium from LPS-stimulated BV-2 cells

**Fig. 5:**
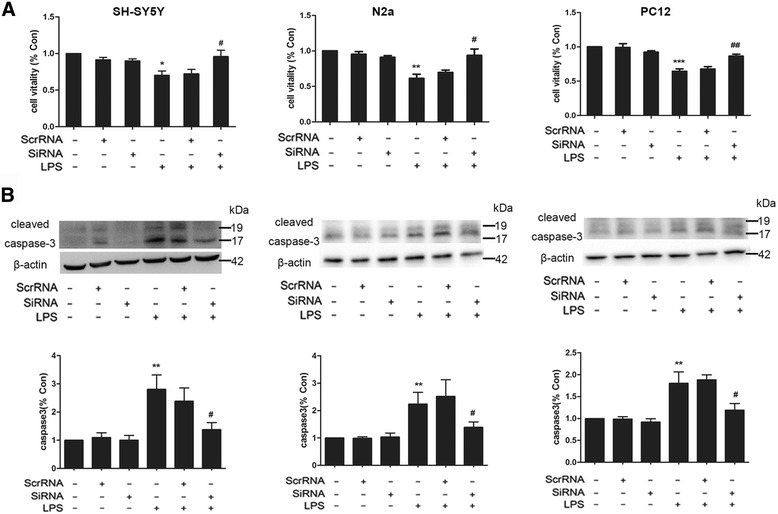
Knockdown of P2Y6R increased neuronal cells viability and attenuated apoptosis when exposed to the conditioned medium from LPS-stimulated BV-2 cells. **a** Effects of the conditioned medium from LPS-stimulated BV-2 cells on cell viability measured by MTT assays. **b** Expression of caspase-3 in neuronal cells (SH-SY5Y, N2a, and PC12 cells), exposed to the conditioned medium from LPS-stimulated BV-2 cells. Data are shown as the mean ± SD of three independent experiments. *p < 0.05, **p < 0.01, ***p < 0.001 vs Con, ^#^ *p* < 0.05, ^##^ *p* < 0.01 vs LPS

To further investigate the effect of P2Y6R in microglia on neuronal cells viability, conditioned medium from LPS-stimulated BV-2 cells was added to different neuronal cells (SH-SY5Y, N2a, and PC12 cells). We transfected the BV-2 cells with P2Y6R siRNA and then incubated the neuronal cells with the supernatant from BV-2 cells treated with LPS. The cell viability and the degree of apoptosis of the neuronal cells were then measured based on MTT assay and the expression of caspase-3. The results showed that the supernatant of the LPS-treated BV-2 cells induced a decrease in cell viability (Fig. 5a) and an increase in caspase-3 expression levels in different neuronal cells (SH-SY5Y, N2a, and PC12 cells) compared with the cells exposed to normal medium (Fig. 5b). However, the decreased cell viability (Fig. 5a) and increased caspase-3 expression (Fig. 5b) were reversed by knocking down P2Y6R. This approach indicated that P2Y6R in microglia contributed to the cell death of neuronal cells and that knocking down P2Y6R could increase cell viability and decrease apoptosis of neuronal cells caused by the cytotoxicity of LPS-stimulated BV-2 cells.

### The activation of P2Y6R is partially dependent on ERK1/2 phosphorylation

**Fig. 6:**
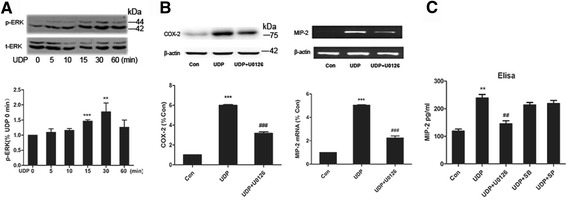
Activation of P2Y6R by UDP is partially dependent on ERK1/2 phosphorylation. **a** Representative western blots showing that ERK1/2 phosphorylation was stimulated in a time-dependent manner by UDP. **b** Representative western blots and RT-PCR showing that COX-2 protein and MIP-2 mRNA levels were decreased in UDP-stimulated BV-2 cells pretreated with U0126 (ERK1/2 inhibitor). **c** ELISA showing that MIP-2 secretion level was decreased in UDP-stimulated BV-2 cells pretreated with U0126. SB (SB203580, P38 inhibitor), SP (SP600125, JNK1/2 inhibitor). Data are shown as the mean ± SD of three independent experiments. *p < 0.05, **p < 0.01, ***p < 0.001 vs Con, ^##^ *p* < 0.01, ^###^ *p* < 0.001 vs LPS

Previous studies have shown that P2Y receptor signaling is mainly through mitogen-activated protein kinases (MAPK) [31, 32]. To explore the mechanism by which P2Y6R is involved in neuroinflammation process, we investigated the levels of MAPK, including the extracellular signal-regulated protein kinase (ERK1/2), p38, and the jun-NH2-kinase (JNK). We found that P2Y6R activation induced by its specific agonist UDP caused strong and rapid ERK1/2 phosphorylation (Fig. 6a). However, UDP had no effect on p38 and JNK expression, whereas LPS could activate all of the MAPK pathways (data not shown). In addition, the involvement of MAPK pathways in P2Y6R activation was also examined using a pharmacological approach. We investigated the impact of specific inhibitors for ERK1/2 (U0126), p38 (SB203580), and JNK1/2 (SP600125) on MIP-2 mRNA expression, COX-2 protein expression, and MIP-2 secretion. Inhibition of the ERK1/2 pathway by U0126 applied 30 min before UDP stimulation resulted in a significant decrease in COX-2 protein and MIP-2 mRNA expression (Fig. 6b). However, no significant inhibition of MIP-2 transcript expression was observed in SB203580- and SP600125-pretreated cells (data not shown). Similarly, pretreatment of the BV-2 cells with U0126 but not SB203580 or SP600125 before UDP stimulation abolished the release of MIP2 measured by ELISA (Fig. 6c). These results suggest that ERK1/2 activation is important in the regulation of P2Y6R cytotoxicity.

## Discussion

Microglia cells are well known as sensors in brain-damaging events and are crucial to the progress of many neurodegenerative diseases [33]. It is now well recognized that microglia functional plasticity is strictly stimuli-dependent; however, how microglia coordinate inflammatory responses is still not clearly understood. In this study, we demonstrated for the first time that UDP/P2Y6R signaling was actively involved in the mechanism of neuroinflammation through an autocrine loop based on LPS-triggered UDP secretion, which resulted in the death of dopaminergic neurons that was partly dependent on the phosphorylation of ERK1/2. Notably, evidence of elevated P2Y6R levels in the PBMCs of sporadic PD patients in China was shown, suggesting that P2Y6R may be a potential biomarker for the diagnosis of PD.

Parkinson’s disease is a neurodegenerative disorder that is estimated to affect more than 1% of the population aged over 65, and there will be more than 10 million PD patients in the world by 2030 [34]. However, to date, the diagnosis of PD is mainly based on clinical manifestations and is confirmed only upon autopsy. Researchers have made many attempts to explore its possible biomarkers and, as a result, some interesting candidates have been identified and proven to be useful alone or in combination [35]. For example, extracellular α-synuclein quantification in human body fluids, such as cerebrospinal fluid (CSF), plasma [36], or saliva [37], has been proposed as a potential biomarker for synucleinopathies. Several studies have reported decreased levels of homovanillic acid (HVA) and DJ-1 in the CSF of PD patients compared with healthy controls [38]. We investigated P2Y6R mRNA levels in PBMCs among PD patients and age- and gender-matched MSA patients and healthy controls. The result showed that P2Y6R mRNA levels in PD patients were significantly higher than in MSA patients and healthy controls. We found that the expression levels of P2Y6R mRNA in PD patients were much higher than in healthy controls among the different age groups, except for subjects over age 80, which may be due to the fewer subjects included in this group. Even though only 30 MSA patients were included in this study, we also found that the P2Y6R mRNA levels in MSA patients was significantly lower compared with PD patients, and the diagnostic specificity was as high as 93.7%, which indicates that the level of P2Y6R in PBMCs might be a specific biomarker for PD and could be used for the differential diagnosis between PD and MSA. Further analysis revealed that increased P2Y6R levels persisted during the entire disease process and was not correlated with disease severity, clinical type, or drug administration, which indicated that P2Y6R mRNA levels cannot be used to monitor disease progression but represent a stable biomarker of PD. Circulating blood cells can contact brain tissue and may provide information reflecting the pathological environment of the PD brain [39]. Compared with other biomarkers, P2Y6R can be measured in the blood and therefore can be easily acquired from patients and tested.

Elevated inflammatory cytokines are found in PD patients’ brains, and inflammation is thought to be a major contributor to the neurodegeneration [4, 40]. In addition, a systemic inflammatory response results in the production of cytokines, which circulate in the blood and communicate with neurons within the brain. It has been demonstrated that peripheral cytokines in PBMCs are increased in patients with PD [41]. Microglia are known as resident macrophages in the central nervous system. PBMCs may provide information reflecting the immune activity of the brain in some extent. The elevation of P2Y6R expression in the PBMCs corresponded to the inflammation of PD patients.

**Fig. 7:**
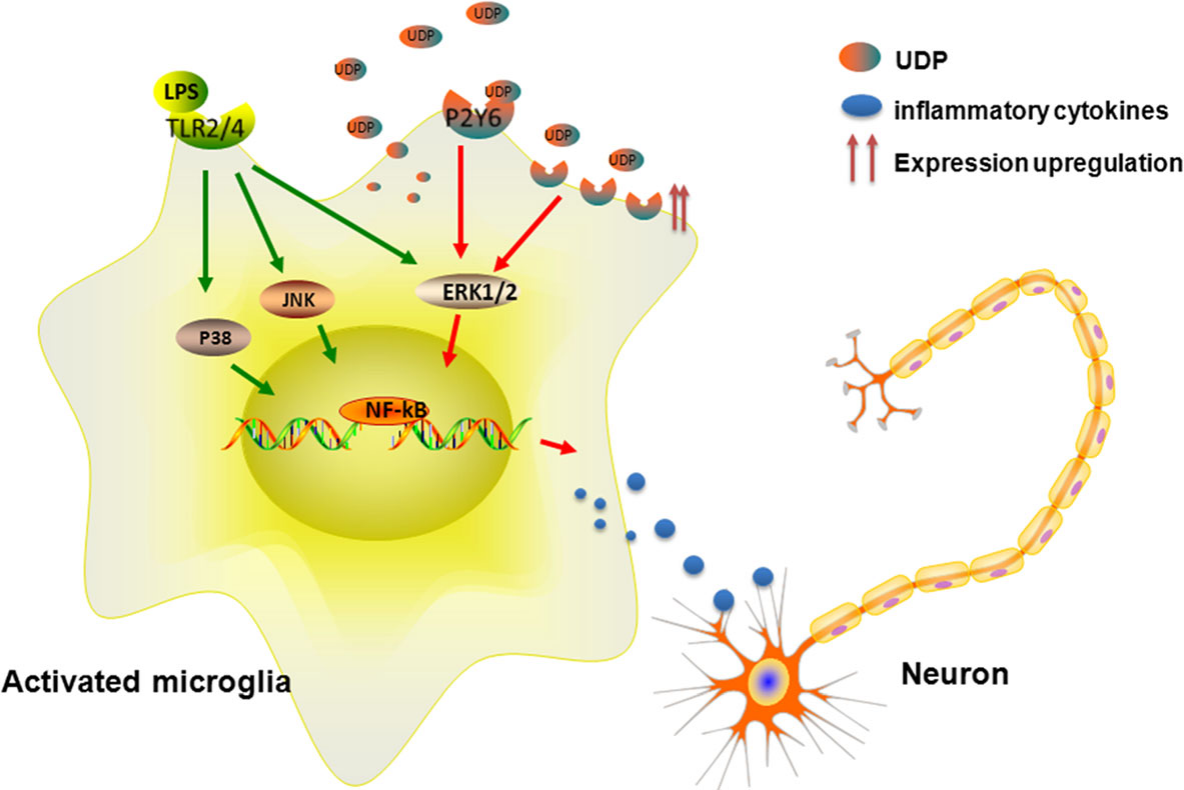
Schematic of UDP/P2Y6R signaling mediating neuroinflammation and inducing the death of neurons

Upregulated P2Y6R expression was confirmed in vitro by inducing an inflammatory stress-like insult with LPS in BV-2 cells. The upregulation of P2Y6R expression following inflammatory stress has been reported in inflammatory bowel disease and in a model of radiation-induced brain injury [19, 42]. We found that UDP/P2Y6R signaling was also involved in neuroinflammation. The mechanism mediating this process is shown in Fig. 7.

We found that activated microglia could upregulate P2Y6R expression. In addition, the change in P2Y6R expression was accompanied by the upregulation of inflammatory cytokines. Thus, we proposed that P2Y6R may participate in the neuroinflammation induced by LPS. UDP was a natural ligand of P2Y6R. It has been demonstrated that injured or damaged neurons release significant amounts of this nucleotide in a paracrine manner, which acts on P2Y6R to initiate microglial phagocytosis [22]. In this study, we found that LPS could trigger an autocrine loop of UDP secretion in activated microglia. The combination of UDP and P2Y6R could then activate downstream mechanisms of the inflammatory response, while either inhibition or knock down of P2Y6R or abrogation of UDP signaling can partially downregulate the expression of the inflammation cytokines induced by LPS. We further revealed a role of P2Y6R in the regulation of microglia function, as it could not only initiate microglia phagocytosis but also actively participate in the process of microglia-induced inflammation.

With respect to the function of P2Y6R, some studies reported UDP/P2Y6R signaling could protect astrocytes against TNF-α induced apoptosis by preventing the activation of both caspase-3 and caspase-8 [43] and could protect mice from vesicular stomatitis virus infection through an increase in IFN-β production [44]. However, other study reported that activated P2Y6R may have a toxic effect, mediating nitric oxide and iNOS production and cause excessive astrogliosis [32]. PD is caused by the loss of dopaminergic neurons, and neuroinflammation is primarily involved in this pathogenesis. Our results demonstrated that LPS could induce the release of many proinflammatory factors from BV-2 and primary microglia cells and that UDP/P2Y6R signaling contributed to this process. We found that conditioned medium from LPS-treated BV-2 cells was toxic and increased the apoptosis of neuronal cells. However, knockdown of P2Y6R in BV-2 cells decreased neuronal apoptosis and increased cell viability, highlighting the concept that activated microglia can damage neurons partly through UDP/P2Y6R signaling. Supporting this viewpoint, we also found decreased MIP-2 release in the conditioned medium from BV-2 cells treated with LPS after inhibition of P2Y6R. We suggest that neuroinflammation mediated by P2Y6R might play an important role in the pathogenesis of PD. This is the first study to explore the relationship between P2Y6R and dopaminergic neurons, emphasizing the effect of inflammatory cytokine production based on interactions with UDP/P2Y6R in the apoptosis of neurons.

We sought to identify the underlying mechanism of P2Y6R activation in accelerating neuroinflammation. Previous studies have found that P2Y receptor binding with the corresponding ligand could regulate activity through a variety of intracellular signal transduction pathways, mainly MAPK signaling pathways [31, 32]. Purine receptors can also activate many transcription factors, including NF-κB, NFAT, CREB, and AP-1 [45–47]. We observed that activation of P2Y6R by UDP could induce the phosphorylation of ERK1/2 but not p38 or JNK. A ERK1/2 inhibitor downregulated the expression levels of MIP-2 and COX-2. A similar phenomenon has been observed in monocytes [28] and intestinal inflammation [17]. It was reported that UTP markedly increased the phosphorylation of ERK1/2, p38, and JNK in cultured spinal microglia [32], while we did not observe the activation of p38 and JNK, which may be due to the different agonists and cell lines used. However, LPS could activate all of the MAPK pathways, and this discrepancy suggested that the effect of LPS on neuroinflammation cannot be solely explained by the release of UDP and the activation of the ERK1/2 pathway via P2Y6R. Nonetheless, our results support a key role of UDP/P2Y6R signaling coupled with ERK1/2 in stimulating the release of inflammatory cytokines. However, the exact mechanism of P2Y6R upregulation in PD patients and its activation in microglia remain largely unknown and warrant further investigation.

## Conclusions

There are two novel aspects of our study: (1) P2Y6R can be used as a potential clinical biomarker of PD and (2) microglial P2Y6R is involved in the neuroinflammation through an autocrine loop based on LPS-triggered UDP secretion and accelerated neuroinflammatory responses through the ERK1/2 pathway. These findings suggest that blocking P2Y6R should be explored as a potential therapeutic strategy for the treatment of PD patients.

## Abbreviations

CNS: Central nervous system
COX-2: Cyclooxygenase-2
CSF: Cerebrospinal fluid
CXCL8: C-X-C motif chemokine ligand 8
ERK1/2: Extracellular signal-regulated protein kinase
HPLC: High-performance liquid chromatography
HVA: Homovanillic acid
H-Y: Hoehn and Yahr
IL-6: Interleukin-6
iNOS: Inducible form of nitric oxide synthase
JNK: Jun-NH2-kinase
LPS: Lipopolysaccharide
MAPK: Mitogen-activated protein kinases
MIP-2: Macrophage inhibitory protein-2
MIP-3a: Macrophage inflammatory protein-3
MSA: Multiple system atrophy
NF-κB: Nuclear factor kappa B
PBMCs: Peripheral blood mononuclear cells
PD: Parkinson’s disease
ROC curve: Receiver operating characteristic curve
TLR: Toll-like receptor
TNF-α: Tumor necrosis factor-α
UDP: Uridine-5’-diphosphate sodium
UPDRS: Unified Parkinson’s Disease Rating Scale
